# From English to “Englishes”: A Process Perspective on Enhancing the Linguistic Responsiveness of Culturally Tailored Cancer Prevention Interventions

**DOI:** 10.2196/57528

**Published:** 2024-12-19

**Authors:** Alexis Davis, Joshua Martin, Eric Cooks, Melissa Vilaro, Danyell Wilson-Howard, Kevin Tang, Janice Raup Krieger

**Affiliations:** 1 University of Florida Gainesville, FL United States; 2 Department of Family, Youth and Community Sciences, Institute of Food and Agricultural Sciences College of Agricultural and Life Sciences University of Florida Gainesville, FL United States; 3 Bethune Cookman University Daytona Beach, FL United States; 4 Department of English Language and Linguistics Institute of English and American Studies, Faculty of Arts and Humanities Heinrich Heine University Düsseldorf Düsseldorf Germany; 5 Mayo Clinic Jacksonville, FL United States

**Keywords:** behavior change, community-engaged research, cancer screening, colorectal cancers, communication, communication accommodation, linguistics

## Abstract

Linguistic accommodation refers to the process of adjusting one’s language, speech, or communication style to match or adapt to that of others in a social interaction. It is known to be vital to effective health communication. Despite this evidence, there is little scientific guidance on how to design linguistically adapted health behavior interventions for diverse English-speaking populations. This study aims to document the strategies used to develop a culturally grounded cancer prevention intervention with the capabilities to linguistically accommodate to speakers of African American English (AAE). We describe the iterative process of developing a cancer prevention intervention with contributions of racially and linguistically diverse colleagues representing various community and institutional perspectives, including communication scientists, linguists, a community advisory board, professional voice talents, and institutional representatives for scientific integrity. We offer a detailed description of the successes and, in some cases, failures of strategies. Social stereotypes associated with AAE were prevalent at both institutional and community levels, resulting in unanticipated challenges and delays during intervention development. The diversity of linguistic, racial, and role identities within the message development team was integral to successfully addressing and identifying opportunities for process improvement. Language is a vital but often overlooked aspect of intervention development. Message designers should consider implicit social stereotypes that unintentionally shape linguistic choices. This study provides a novel overview of how various types of expertise and iterative message development processes contribute to successfully navigating cultural grounding when sensitive or stigmatized issues are salient.

## Introduction

Addressing cancer health inequities, particularly related to screening, is a key public health goal in the United States [1]. One such inequity is the low rates of colorectal cancer screening and the high rates of colorectal cancer mortality among African Americans in the United States [2,3]. There is widespread agreement that these efforts must be developed in partnership with the communities affected to ensure they are culturally grounded, meaning that the intervention honors and reflects salient social identities within a community [4]. Referred to as the principle of cultural grounding [5], this approach uses community engagement processes to codevelop messages that privilege how cultural identities are communicated [6].

Cancer screening messages are typically delivered using standardized forms of language, with English and Spanish being the most common in the United States. There are several reasons to question the appropriateness of defaulting to standardized forms of language in the cancer prevention context. First, the way language is used and spoken varies by region and cultural group [7]. Second, these variations indicate social group identities (or lack thereof) [8]. Third, group memberships serve as cues for status and credibility based on individual position as an ingroup or outgroup member [9]. Fourth, the ability to use language effectively positively influences health outcomes [10]. For example, African American physicians have been found to engage in “code-switching” when interacting with African American patients. This means they use a variety of English called African American English (AAE) to establish rapport and switch to Standard American English (SAE) for medical tasks such as history taking [11]. This example demonstrates that physicians naturally recognize the value of reflecting a shared social group identity through language for improving health outcomes.

The goal of this project is to explore the potential benefits and risks of reflecting these naturalistic communication accommodation patterns between African American physicians and patients in the digital intervention space. The project’s main goal was to develop an evidence-based colorectal cancer prevention intervention delivered in AAE by a virtual health care provider (VHCP) that would be perceived as culturally appropriate and authentic. This will enable future research to test whether having the option of VHCPs who can effectively use AAE could increase colorectal cancer screening among native speakers of this English language variety. To achieve this goal, the research team used the principle of cultural grounding to foster partnership and collaboration with diverse stakeholders. This manuscript describes challenges encountered while navigating perceptual biases associated with AAE and how those challenges were resolved. We hope this study serves as an impetus for wider conversation on the importance of language in developing culturally grounded behavioral interventions.

## Literature Review

### Communication Accommodation Theory

According to communication accommodation theory, humans navigate identity and solidarity with interlocutors through linguistic accommodation [12]. It highlights the ways in which people implicitly and explicitly identify themselves and others during social interactions by modifying various aspects of communication. A key attribute of this theory is that perceived similarity with others influences how people construct and interpret messages. If we feel connected to someone, we speak more like they do; if we feel disconnected, we dissociate our speech from theirs. This is especially true in patient–health care provider communication [13]. A communicative interaction is perceived as interpersonal if the messages exchanged are based on individual characteristics. Alternatively, it can also be experienced as intergroup, meaning that the choice of communication is influenced by salient social identities (eg, race, gender, age) rather than the unique characteristics of the individuals. Even when a patient and health care provider share salient social identities, power differentials based on social status can result in interactions being more intergroup than interpersonal in nature.[14]

When interactions are experienced as intergroup, identity negotiation becomes critically important to the success or failure of that interaction. Language plays an essential role in identity negotiation. Efforts to accommodate to a shared linguistic style—or become more linguistically similar to another person—improve communication effectiveness, understanding, and trust [15]. Both objective and perceived language similarities enhance perceptions of credibility, competence, and persuasiveness [16]. Clinicians and patients alike concur that health care providers should accommodate the linguistic style of patients, despite disagreeing about whether accommodation has occurred [17].

Perceived similarities play a crucial role in the evaluation of health care providers. Indeed, perceived dissimilarity is linked to biases, prejudices, and negative assessments of the person and their interactions [18]. However, accommodation can be challenging in interactions when a provider and patient have different racial identities and have differing levels of skill with AAE. In interactions where health care providers lack the skill to accommodate to AAE, patients may maintain or accentuate linguistic features associated with AAE for the purpose of intergroup differentiation [19]. Conversely, health care providers with the ability to speak both SAE and AAE have more flexibility to accommodate to the speech of diverse African American communities [13,20]. For the purposes of our work here, African American is defined as an individual who self-identifies as Black. Thus, both terms may be used to describe individuals in this study. Even though the use of SAE may be perceived as more common in a medical setting, a patient may find AAE more persuasive because it reflects a shared racial and linguistic identity [21-23].

Following this reasoning, we hypothesize that an African American health care provider using linguistic features drawn from AAE could be viewed as accommodating linguistic styles by African American patients whose own native dialect contains similar features. Indeed, previous research has shown that shared racial identity by visual appearance had a clear positive effect: When African American patients were paired with a virtual health care provider (VHCP) matching their racial identity, they reported higher intentions to be screened for colorectal cancer [24,25]. VHCPs are graphical representations of characters that display human-like behavior and can be effective sources of tailored health information. For interactions between humans and virtual agents, measuring the level of human communication accommodation can aid in understanding how effective virtual agents are at engaging patients in a satisfying and credible manner. VHCPs are a feasible way to experimentally examine how different communication elements influence patient outcomes. Thus, this project sought to examine whether adjusting linguistic features of the VHCP speech will have a positive, neutral, or negative effect.

Adapting the linguistic features of a cancer prevention message can be considered part of message tailoring. Tailored messages use a high level of individual customization, or accommodation, to increase relevance and are an evidence-based strategy for communicating complex health topics with the public [26]. Tailoring allows for the personalization of a message based on specific characteristics of the recipient. In doing so, the message and its content become more personally relevant and more likely to have a lasting impact on attitudes and behaviors [27]. Tailored messages have been found to specifically increase the persuasiveness of messages encouraging colorectal cancer screening, improve attitudes toward screening, and facilitate screening behaviors [28]. Tailored messaging as an intervention strategy is growing increasingly popular due to the combination of efficacy in promoting behavior change and the ability to be produced and disseminated cost-effectively using web-based applications [29].

### African American English

Linguistic investigation into African American language use has increased over the last 60 years [30]. A central objective of this work is to dispel racist linguistic myths, explicate the systematic nature of the linguistic varieties of many African Americans, and dismantle the linguistic discrimination and institutional racism that has produced disparate harm for speakers in realms such as education, job procurement, and health care [31].

Researchers have identified specific pronunciations, sounds, and grammatical structures from the speech of various groups of African Americans across the United States and throughout various periods that do not appear in SAE. Together, these structures, along with other linguistic features, have traditionally been termed AAE [32]. These linguistic features are not inferior or flawed versions of SAE counterparts; they rather display the typical traits of all languages and linguistic varieties and play an important role in social interactions and identity building [33]. However, we note that AAE is not a delimiting set of structures that defines all African American speech. The speech of individual African Americans may or may not contain linguistic features of AAE alongside features of SAE and other language varieties.

The speech of individual African Americans is influenced not only by racialization but also by the intersections of various social identities, such as gender, age, education, sexuality, occupation, religion, ability status, socioeconomic class, and regional background [34]. All of these layers contribute to larger linguistic repertoires that vary by individual and may be consciously or subconsciously drawn upon as African American speakers engage in identification processes in various contexts and social situations [35].

Thus, African American speech is quite diverse. Nevertheless, African American linguists and scholars estimate that between 80% and 90% of African Americans retain and use a selection of the linguistic features of AAE at varying levels as they weave through the various interactions in their lives [36]. We find it plausible, therefore, that many of the African American participants in our research may use AAE linguistic features at some level and may find AAE linguistic accommodation provided by VHCP appealing.

## Ensuring Linguistic Authenticity Using the Principle of Cultural Grounding

As stated, this project’s goal was to develop an evidence-based colorectal cancer prevention intervention delivered by a VHCP that would be perceived as authentic and culturally responsive. We followed the principle of cultural grounding, which uses community-engaged processes through which communities and professionals can collaboratively ensure linguistic authenticity. We did so in 2 primary ways. First, we used a community advisory board (CAB), which brought members of the community together to provide guidance on issues such as research design and administration [37]. The CAB was comprised of 12 people from racially, ethnically, and geographically diverse communities within a largely rural area [38]. CAB members included individuals representing cancer survivors, the faith-based community, cancer prevention nonprofits, and local health care organizations. They were selected, in part, based on their ability to represent one of the priority populations of the project: individuals who identify as rural-dwelling and Black or African American. The collaboration between CAB and the research team helped to ensure the intervention was perceived as appropriate and reflected speech used in the community.

Second, we sought professional voice talent to join our research team. Given that the scripts would be tested in an experimental design, it was imperative that the different script variations be voiced by the same person. After an extensive search, the team hired 2 voice actors who were native speakers of AAE—1 man and 1 woman. The team discussed the project’s goals with the voice talents, who were asked to read the same script three times, each with a different linguistic variation.

## Workflow for Script Development

The idea for this project emerged when several investigators were working on a colorectal cancer prevention project for populations identifying as rural and Black or African American. To advance the dissemination of the intervention, the team began to consider how to use artificial intelligence (AI) to enable speech recognition. One of the principal investigators (PIs) was introduced to a linguist who explained that biases in AI make it difficult to respond to the natural speech of AAE speakers [39]. Given this limitation for a key population, the PI and linguist decided to jointly pursue this project to explore perceptions of linguistic accommodation in cancer prevention interventions among AAE speakers.

The script for the original intervention served as the base script for this project. When the original script was created using SAE, it was reviewed by multiple stakeholders, including CAB members, scientific advisory boards, grant reviewers, and physicians, without any mention of whether it would be appropriate to have linguistic variations of English represented. This points to the ubiquitous expectation of using SAE in cancer prevention interventions. Next, we describe the iterative process we used to create a new script for this project.

### Script Adaptation

The first step for the team was to revise the base script linguistically. The goal was to create 3 scripts: in SAE, low-level African American English (L-AAE), and high-level African American English (H-AAE), with the following breakdown for inclusion of morphosyntactic (ie, grammatical) and phonological (ie, sound) features of AAE (Table 1). The process of adaptation began with the work of a linguist (linguist #1) who was an early career researcher and not a native AAE speaker. It was important that the script reflected AAE accurately without potentially causing offense through vocabulary or grammatical structure. To bolster the script’s authenticity, linguist #1 referred to Green’s *African American English* [33] and Rickford’s *African American Vernacular English: Features, Evolution, Educational Implications* [36]. Both resources were written by African American linguists and detail the many aspects of AAE syntactic and phonological structures.

Linguist #1 began with the base SAE script that had been used in a pilot intervention the year before [24]. They used phonological features such as final consonant cluster reduction and “*-*ing” dropping, as well as morphosyntactic features like the absence of past tense “-s”. These features were added according to the Dialect Density Measure (DDM), which quantifies the number of dialectal features of a speaker to determine “how much” of a dialect the individual speaks. Linguist #1 collected several DDMs: the lowest DDM (least number of dialectical features) came from sampling naturally occurring speech (interviews and focus group discussions with African Americans conducted for a previous project [40]); the other level (highest DDM) came from “Reactions to African American Vernacular English: Do More Phonological Features Matter?” [41]. These DDMs were used to determine how many dialectical features to include in variations of the script. They were particularly important because they provided accuracy in choosing features in the absence of insight from a native AAE speaker. Each feature added to the script was linked to a corresponding audio example contained within the Corpus of Regional African American Language (CORAAL) for further corroboration of the features’ authenticity [42].

**Table 1:** Inclusion of 2 types of African American English linguistic features (morphosyntactic and phonological) in 3 script types (SAE, L-AAE, and H-AAE).

	SAE^a^	L-AAE^b^	H-AAE^c^
AAE^d^ morphosyntactic features	No	No	Yes
AAE phonological features	No	Yes	Yes

^a^SAE: Standard American English.

^b^L-AAE: low-level African American English.

^c^H-AAE: high-level African American English.

^d^AAE: African American English.

### Script Revision

After the 3 scripts were created, we provided the materials to an early career researcher, a linguist who spoke AAE natively (linguist #2 hereafter), and asked them to suggest changes and provide feedback for the L-AAE and H-AAE scripts. Linguist #2’s main priority was to ensure that participants who would hear a script would be able to understand the information conveyed and feel accommodated by the language choices. Linguist #2 wrote a list of suggestions for the L-AAE and H-AAE scripts, as well as their own version of the H-AAE script, using linguist #1’s script as reference (to compare the script crafted with research with one crafted with reliance on a native speaker’s intuition). We provide an example of the differences between the scripts, with the underlined words and phrases indicating AAE features (Multimedia Appendix 1).

### Script Recordings and Finalization

The team then held meetings with the voice actors to discuss the details of the initial recordings. Some rerecordings were made for scripts to address the accidental omission of AAE features on the part of the voice actors. These adjustments were necessary to ensure each version was distinct to avoid confounding the experimental study design. For example, if the voice actors were to use an abundance of H-AAE characteristics in the L-AAE reading, then the team would not be able to determine the effect of the randomized condition. During the rerecordings, some sentences or phrases were corrected for prosodic issues. However, the accuracy of phonological and morphosyntactic features of AAE was prioritized over suprasegmental features.

It was imperative that the voice actors understand the goals of the study, the team’s aspiration for authenticity and accuracy, and the importance of feeling comfortable using “nonstandard” language. Attention was also paid to the possible reception of scripts tailored to African American populations, and several team discussions focused on preventing the potential for activating negative social stereotypes, such as “broken English,” “uneducated,” and “lower class.”

## Insights Gained

### Insights From the Community Advisory Board

Before the script was finalized and the recordings created, the research team held a meeting with the CAB where several concerns, potential benefits, and recommendations were raised, as summarized below. First, the CAB agreed that health terminology in the script should not be changed or “dumbed down” (eg, the term “sugar” should not be used instead of “diabetes”). This concern may have been based on a perception of over-accommodation. That is, using nontechnical terminology could be perceived by African American users as reflecting negative social stereotypes (eg, unintelligent). This mirrors how people tend to use less complex grammatical structures, a more limited vocabulary, and more repetition, simplification, and elaboration when speaking to small children (child-directed speech), adults with limited cognitive or linguistic capacity (eg, elderspeak), and foreigners [43-45].

Second, the CAB expressed how African Americans are often judged by others (including other African Americans) for the way they speak, as either too Black or not Black enough [46]. This led to the question of whether patients would view the VHCP as less professional if it did not use SAE. The board emphasized the importance of authenticity in that the VHCP should sound like how a person would actually speak. If the voice sounded contrived or forced, this could be perceived negatively. Regarding potential benefits, the CAB agreed that linguistic variations might help some patients feel more comfortable in a medical interaction. They mentioned that patients can feel intimidated by health information and health professionals (including community health workers) and that changing linguistic style is, in fact, something community health workers already do to help patients feel more at ease [47]. Therefore, our proposed VHCP intervention is a novel way to improve health knowledge and literacy.

Overall, the CAB viewed the proposed idea as interesting. They made the following recommendations for the project. First, they noted that the study team would benefit by conducting additional research with African American patients to ensure that the developed scripts are authentic and demonstrate cultural sensitivity. Second, the CAB wanted to be involved at each stage of the intervention process. Specifically, they requested to review the recorded voices and the draft scripts to ensure cultural responsiveness. All board members indicated positively that the project could continue to move forward.

### Insights From the Scientific Review Board

As part of the scientific review, an initial reviewer indicated concerns about whether AAE was appropriate for use in an intervention, necessitating 2 rounds of evaluation. Before and during the discussion with the research team, the board was concerned about the study’s potential to be perceived as disrespectful of a cultural dialect or perpetuate stereotypes. Furthermore, the board expressed worry that the study has an underlying premise that all African Americans use or accept AAE.

### Insights From the Linguistics Team

A key goal of this study was to engage the voices of a multidisciplinary and community-engaged research team in all aspects of the study design process. It was important that all team members approved the scripts produced for the intervention. Each team member was interviewed after the project and asked to share their thoughts on the process, their own roles, and any insights into the project.

#### Team Roles

Linguist #1’s roles on the team extended beyond script creation. Originally, they were the point of contact for AAE research and the main creator of stimuli for the original project. That role evolved into becoming project manager, where they began organizing meetings between the researchers and voice actors, liaising with the institutional review board, working on the National Institutes of Health critical review, holding public presentations for the Clinical and Translational Science Institute and the CAB, and playing a role on the grant writing team.

Linguist #2, as the only AAE-speaking linguist on the team, felt pressure to be confident in his decisions during the script-making process and to be accountable for ensuring that the work remained accurate without being offensive. Linguist #2 was the sole AAE-speaking linguist, yet his research background consisted of an in-progress master’s thesis. Thus, it was a struggle to juggle his linguistic expertise with his academic credentials.

Voice actor #1 felt prepared for their role. They had a background in the medical field as administrative support for different professionals and felt comfortable producing voice material for this project because of this previous experience. They enjoyed the ability to be frank about concerns that arose during the initial stages of working on the project. In addition, they believed that the further integration of their role into the team (not simply as a voice actor but being consulted by the linguists during the course of the script recordings and being able to follow along with study results) was professionally gratifying.

Initially, voice actor #2 had some questions about their role in the project and wanted to make sure those questions would be addressed by the team. They were curious to know “how Black” the team would want them to sound during the voice recordings. In previous works, they had been told to “sound Black” without being given further direction, and they had been left to figure out the level of AAE to use on their own. Voice actor #2 did not want the team to shy away from providing guidance on the language they would use. They appreciated that their role in the project did not remain solely as voice actors but that they were also kept up to date with the project regarding follow-ups and published results.

#### Opportunities and Challenges

In the uncharted territory of AAE script creation, the team faced several challenges. Voice actor #1 was initially apprehensive about speaking AAE for this project. This was not something they were used to doing, and they felt that “speaking Black” could come off as a caricature rather than as an authentic representation that would be well-received. They worked with the linguists on this concern to ensure that they could faithfully record the script and not feel uneasy.

The research team also held similar trepidations. Linguist #1, who is White, wanted to ensure that they did not offend the voice actors themselves while detailing the goals of the project and the script. They were aware that AAE could be a source of controversy, and they gave the voice actors many opportunities to share their concerns. In addition, they took suggestions from the voice actors during the script creation process. Throughout, linguist #1 acknowledged their status as a nonnative speaker of AAE. They would defer to the African American voice actors and linguist #2 (also African American) for final judgments.

Voice actor #2 was greatly appreciative of the level of communication during the project, especially regarding the voice recordings for the script. They felt comfortable with the team and appreciated the more casual atmosphere. They enjoyed the specificity of the acting direction provided, including many annotations, which enabled them to do their job well because their previous experiences had not always included such guidance.

## Discussion

### Perceptions of African American English

The use of developing digital health interventions using AAE use in health contexts is complicated. On the one hand, physicians naturally accommodate to AAE speakers in medical contexts, and linguistic accommodation is associated with a host of positive outcomes in health contexts (eg, increased credibility and social influence). On the other, AAE, like all language varieties, differs by region and other factors. Given the high variability within AAE, the goal is to increase perceived accommodation rather than perfect correspondence. The use of AAE is a social identity marker that is often associated with social stereotypes in the United States. These social stereotypes can make it a sensitive topic. For example, members of the CAB and the scientific review board were cautious about ensuring that the research project did not aggravate implicit bias in health care and thus harm the African American community [48]. Team composition was an important cue. These groups preferred hearing about the value of incorporating AAE in health contexts from members of the team who were native speakers of AAE.

### Perceptions of Team Composition

Language is core to identity; thus, all discussions of language required careful negotiation of the identities of the participants. For example, we consider it likely that African American participants might see an African American VHCP using AAE to deliver colorectal cancer prevention messaging as more authentic and accommodating. For this reason, it was important to build a team that included an African American linguist and native AAE speaker for script development and African American voice actors for VHCP production.

Although considering source characteristics are an essential component of social influence, there were 2 instances in which the racial diversity of the study team was not effectively communicated. This, coupled with salient social stereotypes of the term “AAE,” led to concerns about the project among some stakeholders. The current project is led by a multiracial investigative team, with several investigators who self-identify as Black or African American. However, not all investigators were present at 2 key meetings, which had negative outcomes.

The first misstep was with our CAB. The study idea was presented at the first CAB meeting by linguist #1 (who is White). While CAB meetings are often good opportunities for early career researchers to learn skills regarding engaging with community members about science, the salience of perceptual biases associated with AAE made this study’s concept presentation particularly sensitive. CAB members were enthusiastic about developing an intervention customized to the Black residents of the community. They saw value in having the source be a Black VCHP; however, they initially expressed concerns that presenting health information using AAE could trigger implicit bias, specifically social stereotypes of AAE speakers as uneducated. The research team scheduled a second meeting to enable CAB members to listen to the proposed stimulus, which they determined sounded authentic to how some members of the community speak. While the term “AAE” was perceived as potentially concerning, the audible representation was perceived as authentic and appropriate.

The second instance in which team diversity was not appropriately represented was with the scientific review board, which had to approve the study. Two non-Black investigators presented the study at the initial meeting based on their expertise in linguistics and cancer screening. However, given the focus on AAE, the members of the scientific review board noted the lack of representation of Black or African American investigators. Without knowledge of the team’s true composition, the board had concerns about the propriety of non-Black scientists addressing the proposed research questions. A second meeting was scheduled when all members of the investigative team were available to share their perspectives on the importance of understanding how language concordance or discordance may alter cancer prevention outcomes for racial and ethnic minority populations. The team was also able to share the audio recordings of the stimulus, which assuaged concerns. The study was subsequently approved.

### Research Challenges

One of the greater challenges during this project was conveying the meaning of “sounding Black” to members of the team, including the voice actors, the CAB, the institutional review board, and the scientific review committees. The team knew that there are certain linguistic features that listeners use to determine whether a speaker’s racial identity is African American. Despite conducting estimations of AAE density within scripts, we could not be certain what features would result in users perceiving the VHCP as an authentic AAE speaker. However, our rationale was based on the knowledge that African Americans routinely face discrimination in health care settings, and we wanted to explore if linguistic variation in VHCPs might benefit African American patient populations.

### Conclusions and Key Lessons

People accommodate to the speech styles of others as a routine part of everyday life. This process of accommodation indicates liking and facilitates positive interactions. The extent to which AI-assisted interventions can and should reflect the accommodation patterns that characterize human-human interactions is currently unknown. Understanding the answers to these questions will be key to ensuring that cancer screening interventions can be delivered both ethically and efficiently in the future.

The goal of this study was to address one small piece of this larger puzzle by describing a process for designing a VHCP intervention that incorporated the ability to accommodate to the speech of users who speak AAE. While designing this intervention, key lessons were learned. An important lesson learned was the value of applied linguistics to the development and implementation of cancer prevention interventions. Language is core to cultural identity, and a specific focus on speech can ensure that efforts to engage in cultural grounding reflect both the verbal and nonverbal messages being conveyed to audience members. Another important lesson was that linguistic diversity can be a highly sensitive topic. As such, it was necessary that the linguistic diversity present within the research team be made apparent, and that all members of the research team be present when important concepts were being discussed. A third lesson came from including voice talent in the research team; this choice became a vital component of conveying the authenticity of the approach.

Future cancer screening interventions should consider linguistics as an important component of team science. For interventions with a focus on African American or Black populations, it may be helpful to ensure that there is linguistic diversity on the team. Engaging native speakers of AAE as well as experts in AAE is important, as both can provide valuable expertise on when and how to incorporate linguistic diversity. Finally, it will be important for future cancer screening interventions to explore the conditions under which accommodation to linguistic diversity improves or diminishes intervention efficacy.

## Appendix

Multimedia Appendix 1 Script samples from linguist 1 and linguist 2.

## Abbreviations

AAE: African American English
AI: artificial intelligence
CAB: community advisory board
CORAAL: Corpus of Regional African American Language
CTSI: Clinical and Translational Science Institute
DDM: Dialect Density Measure
H-AAE: high-level African American English
L-AAE: low-level African American English
PI: principal investigator
SAE: Standard American English
VHCP: virtual health care provider
