# Supplementary material for: From English to “Englishes”: A Process Perspective on Enhancing the Linguistic Responsiveness of Culturally Tailored Cancer Prevention Interventions
Source: J Particip Med. 2024 Dec 19;16:e57528. doi: 10.2196/57528 (PMC11695973; doi:10.2196/57528)
Supplement: Multimedia Appendix 1 [file jopm_v16i1e57528_app1.pdf]

H-AAE Sample Written by Linguist #1 (11 features)

I'm ALEX. I'm an online health care assistant with UF Health. Iss nice to meet you. Before we get started, you should know dat iss best to finish your appointmen in one sittin,' so start when you have time [drawn out vowel] for your appointmen and some follow-up questions about your experience. When you ready to start, please tap "Les begin!," or tap "Remine me later" and I'll follow up whichu in a bit.

H-AAE Sample Written by Linguist #2 (29 features)

I'm ALEX, a[uh] online health care assistant [wi]/[wif] UF [Healf]. Iss nice t[uh] meet you. 'Fore we g[ih] star'[id], you should know it's bes' t'finish your appoin'ment in one sitting, so [only] start when you have/[got] time for both/bof your appoin'ment [pause], and some follow-up questions 'bout your experience. When y'ready t'start, go 'head /'n'/ tap "Les begin!", or, [you can hit] "Remine me lat[uh]" [pause] 'n' I'll follow up witchu at a lat[uh] point.
